# Risk factors for failed fistula closure in Addis Ababa at fistula centre, central Ethiopia

**DOI:** 10.3389/fgwh.2024.1460227

**Published:** 2024-11-21

**Authors:** Tadesse Mamo Dejene, Asrat Kassaw Belachew, Michael Amera Tizazu, Sadat Mohammed Yesuf

**Affiliations:** Department of Public Health, Asrat Woldeyes Health Science Campus, Debre Berhan University, Debre Berhan, Ethiopia

**Keywords:** fistula, failed fistula closure, Hamlin hospital, Addis Ababa, Ethiopia

## Abstract

**Background:**

Obstetric Fistula leads to fecal and urine incontinence in women and girls. Surgical repair is the cornerstone of care. Failure to repair an obstetric fistula exposes women not only to repeated surgery but also to ongoing incontinence and its sequela, depression, and social exclusion. Which impacts the nation's health intervention programs and socioeconomic burden.

**Objective:**

To determine factors associated with failed fistula closure in women who underwent Fistula Closure at the Hamlin Fistula Center in Addis Ababa, central Ethiopia, between February 10, 2018, and December 28, 2020.

**Methods:**

Women who underwent Fistula Closure between February 10, 2018, and December 28, 2020 were included in a case–control study that was conducted between May and June 2021. In total, 417 study participants (139 cases and 280 controls) were selected using a systematic random sampling technique. Two professional midwife data collectors and one BSC nurse for the supervisory assessment of women's medical cards utilized a structured questionnaire to collect data. SPSS version 25 was used to enter, clean, and analyze the data. During data analysis, bivariate and multivariate regression models were used. A *p*-value of less than 0.05 indicates a significant correlation.

**Results:**

Forty-nine patients (35.3%) and 133 controls (47.8%) who were older than 26 years were at repair while they were (14–19 years old). The factors that contributed to failure of fistula closure included age at repair (14–19 years old) [AOR = 2.1, 95% CI (0.94–4.89)], prior fistula attempts (9.6, rural residence [AOR = 2.69, 95% CI (1.36–5.35)], height <150 cm [AOR = 1.80, 95% CI (0.99–3.59)], labor duration longer than 2 days [AOR = 1.89, 95% CI (0.99–3.59)], delivery by cesarean section [AOR = 1.88, 95% CI (1.04–3.89)], damaged urethra [AOR = 2.02, 95% CI (1.04–3.89)], diameter of fistula >3 cm, mild vaginal scar [AOR = 3.20, 95% CI (1.24–8.29)], moderate and severe vaginal scar [AOR = 5.49, 95% CI (1.92–15.75)], and completion of ANC [AOR = 0.20 (0.11, 0.38)].

**Conclusion and recommendation:**

Age at the time of repair, Residence, Height, duration of labor, mode of delivery, completion of ANC, damaged urethra, fistula diameter >3 cm, previous fistula attempts, and vaginal scar are factors related to failure of fistula closure. By focusing on these areas, we can significantly improve the outcomes for patients undergoing fistula repair and lower the likelihood of failed closures in the future. Community-based health education regarding obstetric fistula and the importance of seeing a doctor as soon as possible during labor to lower the risk of obstructed delivery, boost antenatal care completion, and shorten the length of labor are all necessary to prevent failed fistula closure in comparable patients in the future.

## Introduction

In the genitourinary and gastrointestinal tracts, fistulas are aberrant openings or communications between two epithelial surfaces that can develop after protracted labor obstruction. It can result in a woman's incontinence of urine and/or feces (1, 2). Obstetric fistulas are primarily caused by obstructed labor and primarily affect women who are poor and marginalized in developing nations who do not have access to basic medical care (3, 4). An estimated 2–3 million women, conservatively, suffer from untreated obstetric fistulas, and approximately 50,000–100,000 women are estimated by the World Health Organization (WHO) to suffer from obstetric fistulas each year, with at least 33,000 of these cases occurring in sub-Saharan Africa (2, 5, 6). Women who undergo repeated surgery because of fistula and those who fail obstetric fistula closure experience extra social and financial challenges, which also affect the fistula care program. Furthermore, women who undergo repeated surgery are often at risk of health complications, such as infection, discomfort, sexual dysfunction, and subsequent infertility) (6–8).

The World Health Organization estimates that a high number of women are thought to be living with fistulas, and there are a significant number of new occurrences of the condition every year, and roughly 90% of pregnancies with obstetric fistulas end in stillbirth (2, 8, 9). Compared with women who had successfully healed obstetric fistula repair, those in the obstetric fistula repair failure group were more likely to have depression, posttraumatic stress disorder, somatic complaints, and a lack of social support (10–12). More than 6 million women experience obstructed labor each year; more than 90% of these women reside in the world's poorest regions, where they have limited access to high-quality fistula repair services and emergency obstetric care (13–16). The incidence of obstetric fistula in underdeveloped nations is 0.29 per 1,000 women of reproductive age; In Ethiopia, there were approximately 142,389 cases of obstetric fistula in 2013 (2, 3, 17). The problem of fistula remains underreported and a relatively hidden public health concern from program and policy planners, as well as at all levels of the health system. Social stigma, marital breakup, including divorces and separations; financial loss due to employment insecurity; and reproductive system issues, such as menorrhea and infertility, are examples of physical and psychological morbidity (18, 19). For women with fistulas, becoming continent after repair is a new beginning; however, incorrect fistula closure can result in even more despair and loneliness. Additionally, having a fistula that has not closed requires repeat surgery, which increases the social and financial strain on women in the program and decreases the likelihood that the fistula will close successfully in subsequent attempts at repair (20–22). Out of a total of 272 women, 93 (34.2%), 48 (17.6%), 65 (24%), and 64 (23%) had vesico-vaginal, recto-vaginal, urethra-vaginal, and vesicourethral fistula types, respectively, and roughly 173 (64%) of those with obstetric fistula reported experiencing leakage from the fistula for at least a year in the Rwandan study (18, 23).

Studies carried out in Ethiopia at different periods showed that 35.3% of fistulas in Bahir Dar did not close (6), 28.8% in Southern Ethiopia (24), and 18.2% in Southwest Ethiopia (21). Incontinence, women who have ANC follow-up, Place of delivery, mode of delivery, Length of labor, Height, extensive vaginal scarring, a small bladder, urethral damage, prior fistula closure, the vaginal approach, Patients who took antibiotics before surgery, catheterized for 12–14 days, the infection she had after the surgery, and the condition of her bladder neck are factors related to failed fistula closure (1, 11, 12, 16, 23, 25–36). A large portion of previous research in this area has focused on the prevalence of successful fistula closure rates and the general magnitude of the fistula in their specific areas, not on determinants of failed fistula closure, particularly in the study area.

### Objective

To determine the factors that contribute to fistula closure failure in women who underwent Fistula Closure at the Hamlin Fistula Center in Addis Ababa, Central Ethiopia, between February 10, 2018, and December 28, 2020.

### Methods and study setting

The Addis Ababa Hamlin Fistula Center, established in 1974 by Dr. Catherine and Dr. Reg Hamlin, was where this investigation was conducted. The Hamlin Fistula Center is the second hospital in the world to provide patients who have suffered from obstetric tragedies with therapy that can change their lives. Additionally, it provides training on fistula cases to trainers from both domestic and foreign organizations. It is located in Ethiopia's largest capital city, Addis Ababa, and covers 527 km2. The city has an average elevation of 2,355 km (17,726 ft). There are ten sub-cities and 117 districts in all. It is also the largest city in the country in terms of population, with 4,793,699 residents and a projected growth rate of 4.39% by 2020 (Addis Ababa City Administration).

### Study design and period

After examining the follow-up records and operation registration information for fistula patients, a case–control study design was implemented, with failed fistula closure as the case and successful fistula closure as the control.

### Population

The source population comprised all patients with fistula who received treatment at the Hamlin Fistula Center in Addis Ababa. All women who underwent obstetric fistula closure at the Hamlin fistula facility between February 10, 2018, and December 28, 2020 were included in the study and included as cases. All women who did not experience obstetric fistula failure at the same time as those at the Hamlin fistula center were included in the control group.

### Inclusion and exclusion criteria

#### Inclusion criteria

#### Cases

All patients treated at the Hamlin Fistula Center in Addis Ababa between February 10, 2018, and December 28, 2020, experienced Fistula closure failure.

#### Controls

Women who did not experience obstetric fistula failure within the same period as patients at the Hamlin Fistula Center in Addis Ababa.

#### Exclusion criteria

The study on cases and controls excluded individuals with fistulas other than those resulting from obstetric causes and those with inadequate medical records.

### Sample size determination

The double population proportion formula was used to determine the sample size.

Using a 1:2 ratio = 139 cases and 278 controls,

Total = **417**

### Sampling techniques and procedures

The number of repair surgeries performed in the last 3 years was 1,000, with 216, 360, and 424 surgeries performed in 2018, 2019, and 2020, respectively. Every year, the records of women who underwent fistula closure and failed fistula closure were recorded consecutively. For Sampling, practically, obstetric repaired cases and controls were ordered sequentially in each year of their procedure, then after proportional allocation to size systematic sampling techniques were applied to select the cases and controls from each year at every (*k* = 2) interval. However, the first patient and the first control were chosen randomly, and then each calculated interval was 2 until the desired sample size was reached. Cases and controls were selected from the medical records of the women using a systematic random sampling technique. The first patient was chosen randomly and then every 2 (cases and controls) using a sampling strategy based on the *K* = 2 value calculated interval. A woman's incomplete card (did not finish the questions) was ignored, after which the card was reviewed.

### Case ascertainment and control selection

Cases: A total of 139 patients had failed fistula closure but failed following repair.

Controls: 278 women who successfully healed obstetric fistulas served as controls. These 278 controls were selected using a systematic random sampling technique from their medical records in the same manner as that used for the selection of cases.

• Both case and control data were retrospectively analyzed.
• Exposure information was obtained retrospectively from the women's recorded follow-up history.
• Only health facility-based cases and controls were included (i.e., cases and controls were selected from those who underwent repair at the Hamlin Fistula Center only).

### Data collection procedure

Through document inspection, operation notes, and discharge logbooks, data were collected using a pretested, structured questionnaire that was modified from several studies in the literature. Before data collection, a pretest was conducted on fistula cases at the Addis Ababa Hamline Fistula Center, representing 5% of the sample size. This was done to identify any ambiguities and ensure the consistency of the questionnaire. Any necessary corrections were made. One BSC nurse served as the supervisor, and two professional midwife data collectors collected the data. A list of all patients who underwent surgery was obtained from the annual patient chart and operation registration book. The patients who underwent successful or unsuccessful repairs were further divided into two categories. The structured questionnaire, which includes socio-demographic characteristics of the participants, including weight and height of the women, gynecological characteristics of the women like Parity, duration of labor, and place of delivery, including neonatal outcome, previous vaginal scar, and other characteristics, were assessed through the questionnaire.

### Variables

#### Dependent variable

⮚ Failed Fistula Closure (Yes, No)

#### Independent variable

Sociodemographic variables included age at repair, marital status, residence, level of education, occupation, and height. Gynecological characteristics included ANC follow-up, mode of delivery, duration of labor, and status of vaginal scar. Clinical characteristics included the status of the urethra (damaged urethra), bladder neck, route of repair, presence of incontinence, and obstetric fistula characteristics, including type of fistula, previous fistula attempt, and fistula diameter.

#### Operational definition

Failed Fistula Closure: A woman was considered to have failed fistula closure if the hole was not closed and/or incontinent 21 days after the fistula repair procedure.

The fistula classification is based on the distance of a fistula's distal edge from the external urethral meatus (Goh classification). Type 1 fistula: the distance from the external urethral meatus to the distal margin of the fistula is greater than 3.5 cm; Type 2 the distance is 2.5–3.5 cm, Type 3 1.5–2.5 cm and Type 4 <1.5 cm (21).

#### Data quality assurance

The data quality was ensured before, during, and after collection. To confirm that the questionnaire was understandable, practical, and logically organized, 5% (21) of respondents who had visited the fistula center in a different location were pretested. Based on the pretest results, pertinent adjustments were made. One day of instruction on the procedures, techniques, and strategies for collecting data was given to the supervisor and data collectors. Each questionnaire had a unique code that the investigator used to consistently check the questionnaire's accuracy and consistency.

### Data processing and analysis

The data were imported into SPSS Version 25 (Statistical Package for Social Sciences) for cleaning, recoding, categorization, and analysis. The explanatory variables were reclassified, the preliminary data were cleared of inconsistencies and missing values, and any errors were quickly corrected by tracing back and re-entering the original coded data. The data analysis yielded means and standard deviations for continuous variables, and frequencies and percentages for categorical variables.

Univariate analysis was used to calculate descriptive statistics such as the mean, frequency, and percentage, by separately examining each variable. Bivariable analysis was used to examine the link between OF Failed Fistula Closure and each of the independent categorical factors using the most widely used test of association, the chi-square test for categorical variables, and the mean and standard deviation for continuous variables. The crude odds ratio (COR) was calculated to assess each independent variable's correlation with the outcome variable. Variables from the bivariate analysis with a *P* value of 0.20 were included in the multivariate model. The binary logistic regression model was used for the analysis of the categorical outcome variable to identify the determinants of failed obstetric fistula. The outcome variable in this case of failed fistula closure was categorized as (yes or no) after that each independent variable was tested for the presence of a significant association or not.

Questionnaires were reviewed for completeness, and attempts were made to eliminate human and recording errors during data collection by employing exclusion criteria for individuals with errors in recorded data to minimize the missing value of the data. Because the missing values were 5%, a full case analysis was performed for any missing data that arose following this procedure.

The adjusted odds ratio (AOR) and related 95% confidence intervals were derived from the multivariate model. Finally, the Enter approach was used to conduct multivariate logistic regression. The degree of relationship was assessed using the adjusted odds ratio, and the presence of a statistically significant difference between the explanatory and outcome variables was assessed using a *p*-value < 0.05 and a corresponding 95% confidence interval.

## Results

In total, 278 controls, 139 cases, and 417 women participated in the study. The patient's medical card and surgical registration book were used to collect data.

### Sociodemographic characteristics

Regarding the employment status of the participants, there were 52 cases (37.4%) and 124 controls (44.6%), and among the farmers, there were 71 cases (51.1%) and 138 controls (49.6%). A total of 35 (25.2%) women aged 14–19 years and 34 (12.2%) controls were older than the 26-year-old 133 (47.8%) controls, and 49 (35.3%) patients were present during the appointment for fistula repair surgery. Eighty (57.6%) patients and 197 (70.9%) controls were married, and among the women who underwent fistula repair, 110 (79.1%) patients and 176 (63.3%) controls lived in rural areas. Eighty-nine patients who underwent surgery to fix fistulas (64%) and 171 controls (61.5%) were illiterate. During the repair procedure, 144 (51.8%) controls and 84 (60.4%) women weighed 50 kg (Table 1).

**Table 1:** Descriptive statistics on the sociodemographic characteristics of the study participants at Addis Ababa Hamlin Fistula hospital in 2021.

Variables		Failed fistula closure		
		Control (No = 0) (278)	Cases (Yes = 1) (*n* = 139)	Total (*N* = 417)
Age at repair	14–19 year	34 (12.2%)	35 (25.6%)	69 (16.5%)
	20–25 year	111 (39.9%)	55 (39.6%)	166 (39.8%)
	≥26 year	133 (47.8%)	49 (35.3%)	182 (43.6%)
Residence	Rural	176 (63.3%)	110 (79.1%)	288 (68.6%)
	Urban	102 (36.2%)	29 (20, 9%)	131 (31.4%)
Marital status	Married	197 (70.9%)	80 (57.6%)	227 (66.7%)
	Single	31 (11.2%)	19 (13.7%)	50 (12.0%)
	Divorced	50 (18.0%)	40 (28.8%)	90 (21.6%)
Economics status	Low income	62 (22.3%)	43 (30.9%)	105 (25.2%)
	Middle (+)	216 (77.7%)	96 (69.1%)	312 (74.8%)
Educational status	Illiterate	171 (61.5%)	89 (64%)	260 (62.4%)
	Primary and (+)	107 (38.5%)	50 (36%)	157 (37.6%)
Occupation	Housewife	124 (44.6%)	52 (37.4%)	176 (42.2%)
	Daily labor	6 (2.2%)	10 (7.2%)	16 (3.8%)
	Self-employed	10 (3.6%)	6 (4.3%)	16 (3.8%)
	Farmer	138 (49.6%)	71 (51.1%)	209 (50.1%)
Weight of women	<50 kg	144 (51.8%)	84 (60.4%)	228 (54.7%)
	≥50 kg	134 (48.2%)	55 (39.6%)	189 (45.3%)
Height of women	<150 cm	99 (35.6%)	71 (51.1%)	170 (40.8%)
	≥150 cm	179 (64.4%)	68 (48.9%)	247 (59.2%)

### Gynecological characteristics

Forty-five women of cases (32.4%) and 86 controls (30.9%) were primiparous. Regarding the delivery method, SVD was used in 62 (45.3%) cases and 160 (57.6%) controls, whereas C/S was used in 76 (54.7%) cases and 118 (42.4%) controls. In total, 13.2% of the controls and 12.5% of the patients were affected. In addition, 99 patients (71.2%) and 127 controls (45.7%) had complete ANC follow-up data, whereas 40 patients (28.8%) and 151 controls (54.3%) had complete ANC follow-up data.

Regarding the duration of labor, 51 patients (36.7%) and 70 controls (25.2%) both experienced labor that exceeded two days. Among the neonatal outcomes at delivery, 276 (99.3% of controls) and 134 (96.3% of cases) were stillborn. Seventy-two (51.8%) cases and 139 (50%) controls of the women gave birth in a hospital; the remaining 49 (35.3%) women and 86 (30.9%) controls gave birth at home (Table 2).

**Table 2:** Statistics on the gynecological characteristics of the study participants at Addis Ababa Hamlin Fistula hospital, 2021.

Variables		Failed fistula closure		
		Control (No = 0) (278)	Case (Yes = 1) (*n* = 139)	Total (*N* = 417)
Parity of women	One	86 (30.9%)	45 (32.4%)	131 (31.4%)
	Two	66 (23.7%)	42 (30.2%)	108 (25.9%)
	Three	56 (20.1%)	24 (17.3%)	80 (19.2%)
	Four	37 (13.3%)	13 (9.4%)	50 (12%)
	Five and above:	33 (11.9%)	15 (10.3%)	48 (11.5%)
Duration of labor	<2 days	208 (74.8%)	88 (63.3%)	296 (71%)
	≥2 days	70 (25.2%)	51 (36.7%)	121 (29%)
Delivery place	Health center	53 (19.1%)	18 (12.9%)	71 (17%)
	Home	86 (30.9%)	49 (35.3%)	135 (32.4%)
	Hospital	139 (50%)	72 (51.8%)	211 (50.6%)
Mode of delivery	SVD	160 (57.6%)	63 (45.3%)	223 (53.4%)
	C/S	118 (42.4%)	76 (54.7%)	194 (46.5%)
Neonatal outcome	Live birth	2 (0.7%)	5 (3.6%)	7 (1.7%)
	Stillbirth	276 (99.3%)	134 (96.4%)	410 (98.3%)
Vaginal scar	No scar	47 (16.9%)	7 (5%)	54 (12.9%)
	Middle scar	176 (63.3%)	80 (57.6%)	256 (61.4%)
	Moderate and severe scars	55 (19.8%)	52 (37.4%)	107 (25.7%)
Complete ANC is performed	Yes	151 (54.3%)	40 (28.8%)	191(45.8%)
	No	127 (45.7%)	99 (71.2%)	226 (54.2%)

### Clinical and obstetric characteristics

**Figure 1:**
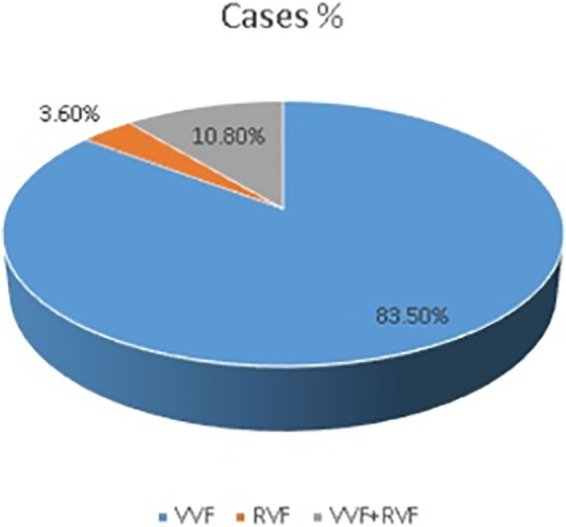
Fistula tear among patients in the Addis Ababa fistula center, 2021.

**Figure 2:**
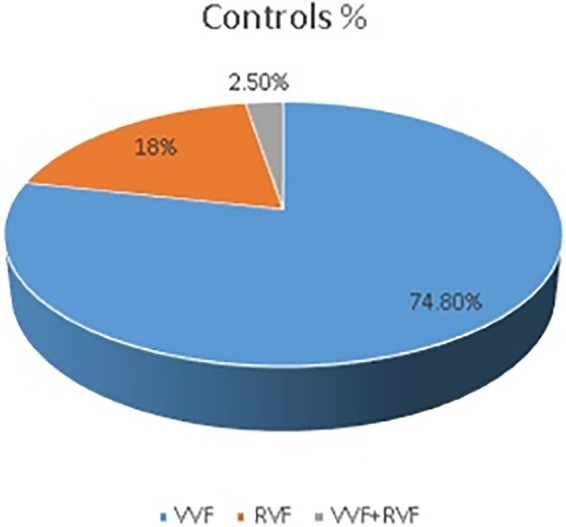
Tear among controls in the Addis Ababa fistula center, 2021.

There were 116 (83.5%) cases and 208 (74.8%) controls who had VVF, whereas 15 (10.8%) cases and 7 (2.5%) controls had both VVF and RVF (Figures 1, 2).

**Figure 3:**
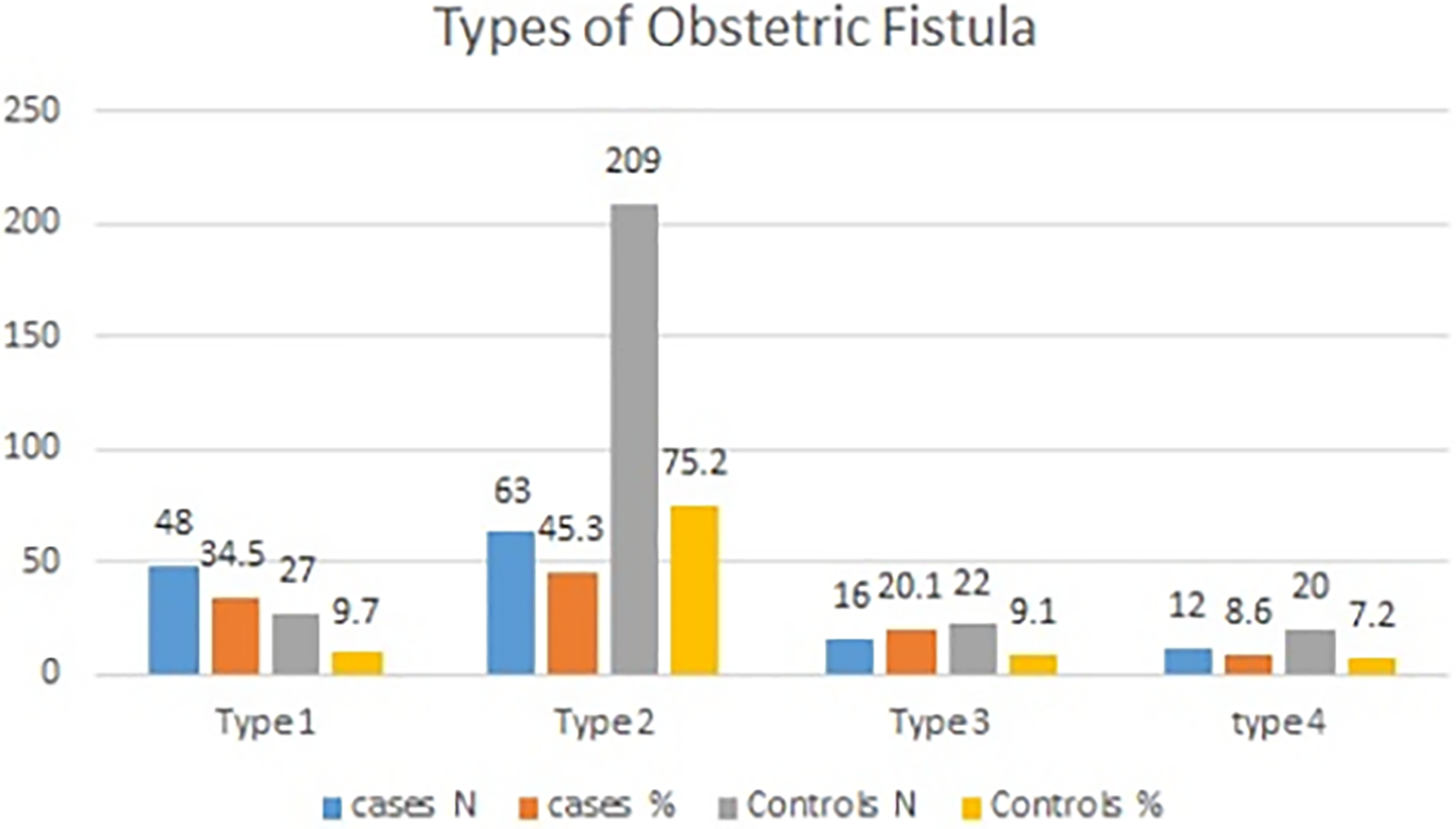
Classification of obstetric fistulas in the Addis Ababa Hamlin Fistula center.

The fistula diameter in 83 (59.7%) patients and 60 (21.6%) controls was >3 cm. Sixty-three (45.3%) patients and 209 (75.2%) controls had type 2 fistulas (Figure 3).

**Figure 4:**
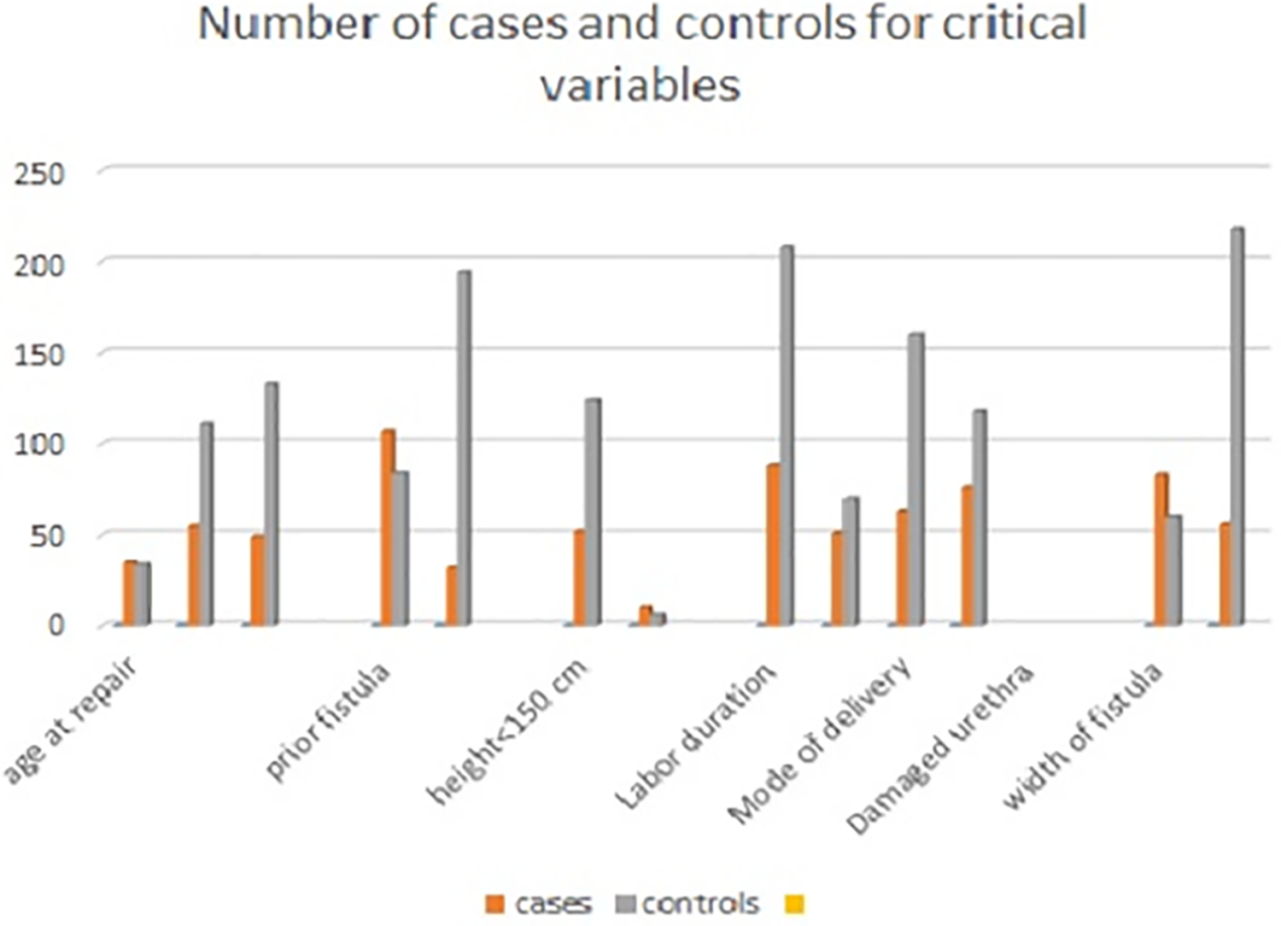
Number of cases and controls for critical variables of obstetric fistula in Addis Ababa, 20.

**(**Sixty-three of the cases were delivered by SVD, and 76 were delivered by C/S (Figure 4). In total, 211 (79.9%) controls and 105 (75.5%) patients underwent reconstructive surgery performed by gynecologists. Ninety-four (67.6%) patients who underwent OF repair developed an infection following the procedure (Table 3).

**Table 3:** Descriptive statistics on the clinical and obstetric characteristics of the study population at Addis Ababa Hamlin Fistula hospital, 2021.

Variables		Failed fistula closure		
		Control (No = 0) (*n* = 278)	Case (Yes = 1) (*n* = 139)	Total (*N* = 417)
Fistula tear	VVF	208 (74.8%)	116 (83.5%)	324 (77.7%)
	RVF	50 (18%)	5 (3.6%)	55 (13.2%)
	VUF + VVF	13 (4.7%)	3 (2.2%)	16 (3.8%)
	VVF + RVF	7 (2.5%)	15 (10.8%)	22 (5.3%)
	Type 1	27 (9.7%)	48 (34.5%)	74 (17.7%)
Type of fistula	Type 2	209 (75.2%)	63 (45.3%)	296 (71%)
	Type 3	22 (7.9.1%)	16 (20.1%)	38 (9.1%)
	Type 4	20 (7.2%)	12 (8.6%)	32 (7.6%)
Previous fistula attempt	Yes	84 (30.2%)	107 (77%)	191 (45.8%)
	No	194 (69.8%)	32 (23%)	226 (54.2%)
Status of urethra	Intact	187 (67.3%)	61 (43.9%)	248 (54.5%)
	Partially damaged	91 (32.7%)	78 (56.1%)	169 (40.5%)
Status of bladder	Intact	202 (72.7%)	67 (48.2%)	269 (64.5%)
	Partially damaged	76 (32.7%)	72 (51.8%)	148 (35.5%)
Presence of incontinence	Yes	278 (100%)	139 (100%)	417 (100%)
	No			
Infection after repair	Yes		94 (67.6%)	94 (22.5%)
	No	278 (100%)	45 (32.4%)	323 (77.5%)
Technique used for failed obstetric fistula closure	Trans vaginal	264 (95%)	132 (95%)	396 (95%)
	Trans abdominal	14 (5%)	7 (5%)	21 (5%)
Repair of the fistula	Senior surgeon	55 (19.8%)	5 (3.6%)	60 (14.4%)
	Gynecologists	211 (75.9%)	105 (75.5%)	316 (75.8%)
	Internist	12 (4.3%)	29 (20.9)	41 (9.8%)
Diameter of the fistula	>3 cm	60 (21.6%)	83 (59.7%)	143 (34.3%)
	<3 cm	218 (78.4%)	56 (40.3%)	274 (65.4%)

### Determinants of fistula closure failure

In the bivariate analysis, the statistical relationships between the independent variables and the outcome variables of fistula-failed obstetric Fistula Closure were evaluated using the crude odds ratio (COR) with a 95% confidence interval. A *p*-value of less than 0.20 indicated that the variable was significant. Thus, failed fistula closure was statistically connected to several sociodemographic traits, including height, residency, and age at repair. Obstetric and clinical features included the following: full ANC follow-up, history of fistula attempt, fistula hole width, bladder and urethra status, and hole width.

Gynecologic traits such as length of labor, delivery method, and vaginal scarring status. However, there was no statistically significant correlation between the outcome variable and the type of fistula, grade, marital status, occupation, economic position, weight, educational status, place of delivery, parity, infection following repair, or technique used to fail the repair (Table 4).

**Table 4:** Determinants of failed fistula closure among women who underwent failed Fistula closure from 2018 to 2020 at the Hamlin Fistula center, Addis Ababa.

Variables		Failed fistula closure				
		Case *N*	Control *N*	COR, 95% CI	AOR, 95%CI	*P* value
Age at repair In year	14–19	35	34	2.80 (1.57, 4.96)*	2.14 (0.93, 4.89)	0.040**
	20–25	55	111	1.35 (0.84, 2.13)*	0.813 (0.42, 1.57)	0.538
	≥26	49	133	1		
Residence	Rural	110	176	2.20 (1.37, 3.54)*	2.69 (1.36, 5.35)	0.004**
	Urban	29	102	1		
Height of women	<150 cm	52	124	2.00 (1.25, 2.85)*	1.8 (1.00, 3.25)	0.049**
	>150 cm	10	6	1		
Duration of labor	<2 days	88	208	1		
	>2 days	51	70	1.72 (1.11, 2.80)*	1.89 (0.99, 3.59)	0.028**
Mode of delivery	SVD	63	160	1.64 (1.09, 2.46)*	1.88 (1.03, 3.59)	0.039**
	C/S	76	118	1		
Previous fistula attempts	Yes	107	84	7.72 (4.83, 12.36)*	7.79 (4.25, 14.29)	0.000**
	No	32	194	1		
Diameter of the fistula	≥3 cm	83	60	5.38 (3.46, 8.39)*	4.96 (2.71, 9.12)	0.000**
	<3 cm	56	218	1		
Vaginal scarring	No scar	7	47	1		
	Mild	80	176	0.16 (0.06, 0.38)*	3.20 (1.24, 8.29)	0.017**
	Moderate–severe	52	55	0.48 (0.30, 0.76)*	5.49 (1.92, 15.75)	0.002**
Complete ANC follow-up	Yes	40	151	1		
	No	99	127	0.340 (0.228, 0.53)*	0.20 (0.11, 0.38)	0.000**
Status of the urethra	Intact	61	187	1		
	Partially damage	78	91	0.38 (0.25, 0.58)*	2.02 (1.04, 3.89)	0.037**

* Significant at *p* < 0.2.

** Significant at *p* ≤ 0.05, *N* = Number.

### Sociodemographic determinants of fistula closure failure

Age at repair has been identified as a significant predictor of fistula Failed obstetric Fistula Closure; women aged 14–19 years had a twofold greater risk of obstetric fistula failure than their counterparts [AOR = 2.14, 95% CI (0.93–4.89)].

Another factor contributing to obstetric fistulas is residence, Failed Fistula Closure, indicating that patients in rural areas are 2.7 times more likely than those in urban areas to experience a failed fistula after pregnancy [AOR = 2.69, 95% CI (1.36–3.35)].

A woman's height was also a determining factor for failed fistula closure in pregnancy; women 150 cm were 1.8 times more likely than their counterparts (those who were more than 150 cm in height) to experience mending failure [AOR = 1.80, 95% CI (1.00–3.24)]. However, multivariate logistic regression revealed that other variables that were substantially linked with obstetric fistula failure were no longer significantly associated (Table 4).

### Gynecological determinants of failed obstetric fistula closure

Women who had mild scarring were three times more likely to experience obstetric fistula failure than those with no vaginal scar [AOR = 3.20, 95% CI (1.24–8.29)]. Moreover, obstetric fistula failure was five times more common in women with moderate and severe scars than that of no vaginal scar [AOR = 5.49, 95% CI (1.92–15.75)].

In addition to these factors, the duration of labor has a substantial impact on obstetric fistula closure. Women who labored for more than 2 days were twice as likely to experience failed fistula closure [AOR = 1.89, 95% CI (0.99–3.59)]. Compared with those who delivered via cesarean section, women who delivered via SVD were twice as likely to experience obstetric fistula closure [AOR = 1.88, 95% CI (1.03–3.59)] (Table 4).

### Clinical and obstetrics determinants of fistula closure failure

Apart from these factors, the urethral status also had a noteworthy impact on the incidence of fistulas, specifically failed fistula closure. This finding implies that women with partially or completely damaged urethras were twice as likely to experience this condition as women with intact urethras [AOR = 2.02, 95% CI (1.04–3.89)].

The other determinant of obstetric fistula failure was the fistula diameter. Women with a fistula diameter of at least 3 cm were five times more likely to experience failed fistula closure than those with a fistula diameter of less than 3 cm [AOR = 4.96, 95% CI (2.71–9.12)].

The likelihood of obstetric fistula failure was seven times greater in women who had attempted fistulas in the past than in those who had not [AOR = 7.79, 95% CI (4.23–14.29)].

All women with ANC follow-up data who experienced obstetric fistulas and who experienced failed fistula closure had a 17% lower rate of incomplete ANC follow-up (Table 4).

## Discussion

Using patient data from the 3-year follow-up after obstetric fistula treatment at the Hamlin Fistula Center in Addis Ababa, this study assessed the factors associated with failed fistula closure. Several factors were found to be significant predictors of Failed Fistula Closure, including age at the time of repair, place of residence, height, urethral status, fistula diameter, completion of ANC follow-up, previous attempts at fistula repair, length of labor, mode of delivery, and vaginal scarring.

Because of issues with fistula closure, women aged 14–19 years are twice as likely to experience failed obstetric fistula closure than those aged over 26 years. Age at repair is a significant indicator of this illness.

Residence is a significant predictor of failed obstetric fistula closure, and rural women are 2.6 times more likely than urban women to experience Fistula Closure failure. This result is consistent with earlier research conducted in Somaliland and Gondar (2, 37).

Individuals who were shorter than 150 cm had a twice-higher risk of failing to achieve successful obstetric fistula closure than those who were taller than 150 cm (38).

Women who had moderate or severe scarring were five times more likely to experience failed obstetric fistula closure than those with significantly different vaginal scar statuses. In a similar vein, women with middle scars were three times more likely to experience obstetric fistula failure than those with mild scars. Other investigations conducted in Guinea in 2016 supported this finding. This national investigation revealed that women with scars had a twofold increased risk of failed fistula closure during pregnancy (19, 20, 22).

Another factor contributing to obstetric fistulas was the diameter of the fistula. Failed fistula closure represents a fivefold increased risk in women whose fistula width is greater than or equivalent to 3 cm. Compared to the 2018 study conducted in Bahir Dar, this result was higher. Women with a fistula diameter of at least 3 cm were twice as likely to have a fistula diameter of less than 3 cm (6, 21, 22, 24, 32).

Apart from these factors, the urethral status also had a noteworthy impact on the incidence of fistulas, specifically failed fistula closure. This finding implies that women with partially or completely damaged urethras were twice as likely to experience this condition as women with intact urethras. The percentage of women with injured urethras was lower in this study than in the Guinea study from 2016, which revealed that women were 5.9 times more likely to have a completely damaged urethra than those who have an intact urethra. The finding of our research is consistent with others who reported that the urethral status of the patient is highly associated with the outcome of interest in this case of failed fistula closure (6, 20–22, 24).

Women who had previously attempted obstetric fistulas were seven times more likely to fail than those who had never attempted fistulas. Furthermore, a 2017 study conducted in Kenya revealed a lower percentage of women than in my study who had attempted repair in the past. Women in this nation who had previously attempted fistulas were 2.9 times more likely to experience failed fistula closure during pregnancy than those who had not (11, 20, 22, 23).

After the ANC follow-up, women who experienced obstetric fistula failure due to Fistula closure decreased by 20% compared with those who did not complete the ANC follow-up. This finding was supported by a study performed in Gondar (2).

This study revealed that labor length had a substantial impact on obstetric fistulas in addition to other factors. Failed Fistula Closure: Women who labored for longer than 2 days were twice as likely to experience this problem as those who labored for less than 2 days. The results were supported by the Bahir–Dar study (6, 22, 24, 38).

This study revealed that the mode of delivery affected the risk of obstetric fistula. Women who had failed fistula closure and delivered SVD were twice as likely to have delivered SVD as those who had delivered c/s. Given that most of the women in this study had labor lasting longer than two days before the women were referred for c/s. A 2016 study conducted in Guinea provides evidence for this conclusion. Women who had delivered SVD were 1.9 times more likely to have done so by c/s in this country study (19, 38).

Our research revealed several factors that were connected to the main complication of unsuccessful fistula repair. These included variables like age at repair, prior fistula attempts, duration of labor, fistula size, and the existence of urethral damage. Furthermore, several important elements that may be involved in the failure of obstetric fistula healing have been highlighted in previous studies. One important factor to consider is the patient's home. Individuals residing in isolated or rural places could find it difficult to receive timely and quality medical care. Another important consideration is the location of the delivery; home deliveries or deliveries to facilities with inadequate equipment are frequently linked to increased risk of problems. Malnutrition is also a serious issue that has been reported by previous studies since it might hinder the body's capacity to mend and recover following surgical treatments. Malnutrition might be another risk factor for failed fistula repair (21). The results of this study can be useful for service providers, especially those working in the study area, because understanding the key variables that contribute to unsuccessful fistula closure can play a role in treating patients with obstetric fistula in clinic settings.

### Strengths and limitations of the study

The study addresses a critical public health issue in Ethiopia, providing valuable insights into the factors associated with failed fistula repair, which is a relatively under-researched area, and identifying factors that have a significant role in the failure of fistula closure, which in turn helps for the intervention to alleviate the problem. A rigorous analysis is another strength of this study. This study has some limitations, including the use of secondary data, which is a limitation of the study. Thus, improved designs will be used in future research in this field to investigate the causes and effects of failed obstetric fistula repair. Although it is the largest and oldest fistula center in the country; One potential limitation of the study is its reliance on data from one institution, the Addis Ababa Fistula Center. To enhance generalizability, it would be preferable to broaden the study to include data from national levels.

## Conclusion and recommendations

Age at the time of repair, Residence, height 150 cm, duration of labor, mode of delivery, Completion of ANC, damaged urethra, diameter of fistula >3 cm, prior fistula attempts, and vaginal scar are all linked to Failed Fistula Closure. Focusing on the interventions of these identified variables, we can significantly improve the outcome for patients undergoing fistula repair and lower the likelihood of failed closure in the future. Community-based health education regarding obstetric fistulas and the importance of visiting a doctor as soon as possible during labor help reduce the risk of developing obstructed delivery. Boosting antenatal care completion, shortening the length of labor, preventing complications during repair procedures, and evaluating patients with senior surgeons before and after the operations are all necessary to prevent failed fistula closure in comparable patients in the future. In addition, the Addis Ababa Health Bureau and other stakeholders should take these factors into account when deciding to intervene in the problem. Finally, future research should focus on cause-effect analysis of failed fistula closure with a better study design to explore the causes of failed fistula closure in this area.

## Data Availability

The original contributions presented in the study are included in the article/Supplementary Material, further inquiries can be directed to the corresponding author.

## References

[1] WHO. Obstetric Fistula: Guiding Principles for Clinical Management and Programme Development. Switzerland: World Health Organization (2006). Available online at: https://www.who.int/publications/i/item/9241593679.

[2] Yismaw L Alemu K Addis A Alene M. Time to recovery from obstetric fistula and determinants in Gondar university teaching and referral hospital, northwest Ethiopia. BMC Women’s Health. (2019) 19:1–8. 10.1186/s12905-018-0700-3 30616532 PMC6323782

[3] Tebeu PM De Bernis L Doh AS Rochat CH Delvaux T. Risk factors for obstetric fistula in the far north province of Cameroon. Int J Gynaecol Obstet. (2009) 107(1):12–5. 10.1016/j.ijgo.2009.05.019 19589525

[4] Maulet N Keita M Macq J. Medico-social pathways of obstetric fistula patients in Mali and Niger: an 18-month cohort follow-up. Trop Med Int Health. (2013) 18(5):524–33. 10.1111/tmi.12086 23489380

[5] Asfaha BT Gebremariam SH Gebremariam GK Weldemariam AG. Knowledge about obstetric danger signs and related factors in reproductive-age women in the southeast zone of Tigray, 2021: a cross-sectional study. Int J Reprod Med. (2022) 2022(1):7346618. 10.1155/2022/7346618 35692452 PMC9177252

[6] Aynie AA Yihunie AG Munae AM. Magnitude of repair failure and associated factors among women undergone obstetric fistula repair in Bahir Dar Hamlin fistula center, Amhara region, northwest Ethiopia. Int J Sci Rep. (2019) 5(11):324. 10.18203/issn.2454-2156.IntJSciRep20194648

[7] Gutman R Dodson J Mostwin J. Complications of treatment of obstetric fistula in the developing world: gynatresia, urinary incontinence, and urinary diversion. Int J Gynaecol Obstet. (2007) 99:S57–64. 10.1016/j.ijgo.2007.06.027 17803995

[8] Kayondo M Wasswa S Kabakyenga J Mukiibi N Senkungu J Stenson A Predictors and outcome of surgical repair of obstetric fistula at a regional referral hospital, Mbarara, western Uganda. BMC Urol. (2011) 11:1–9. 10.1186/1471-2490-11-23 22151960 PMC3252285

[9] Cowgill KD Bishop J Norgaard AK Rubens CE Gravett MG. Obstetric fistula in low-resource countries: an under-valued and under-studied problem – systematic review of its incidence, prevalence, and association with stillbirth. BMC Pregnancy Childbirth. (2015) 15:1–7. 10.1186/s12884-015-0592-2 26306705 PMC4550077

[10] Hancock B Browning A. Practical obstetric fistula surgery. (2009).

[11] Holme A Breen M MacArthur C. Obstetric fistulae: a study of women managed at the Monze mission hospital, Zambia. BJOG. (2007) 114(8):1010–7. 10.1111/j.1471-0528.2007.01353.x 17506793

[12] Adler A Ronsmans C Calvert C Filippi V. Estimating the prevalence of obstetric fistula: a systematic review and meta-analysis. BMC Pregnancy Childbirth. (2013) 13:1–14. 10.1186/1471-2393-13-1 24373152 PMC3937166

[13] Neilson J Lavender T Quenby S Wray S. Obstructed labour: reducing maternal death and disability during pregnancy. Br Med Bull. (2003) 67(1):191–204. 10.1093/bmb/ldg018 14711764

[14] Ezeonwuka I-F. Historical overview of maternal, newborn and child health challenges in a developing economy: the case of Nigeria. Int J Res. (2015) 2(12):725–39. http://eprints.gouni.edu.ng/id/eprint/1999

[15] Ruder BJ. Shattered lives: understanding obstetric fistula in Uganda. (2012).

[16] AbouZahr C. Global burden of maternal death and disability. Br Med Bull. (2003) 67(1):1–11. 10.1093/bmb/ldg015 14711750

[17] Umoiyoho AJ Inyang-Etoh EC Abah GM Abasiattai AM Akaiso OE. Quality of life following successful repair of vesicovaginal fistula in Nigeria. Rural Remote Health. (2011) 11(3):102–8. 21905761

[18] Egziabher TG Eugene N Ben K Fredrick K. Obstetric fistula management and predictors of successful closure among women attending a public tertiary hospital in Rwanda: a retrospective review of records. BMC Res Notes. (2015) 8:1–7. 10.1186/s13104-015-1771-y 26654111 PMC4676892

[19] Delamou A Delvaux T Beavogui AH Toure A Kolié D Sidibé S Factors associated with the failure of obstetric fistula repair in Guinea: implications for practice. Reprod Health. (2016) 13(1):135. 10.1186/s12978-016-0248-3 27821123 PMC5100224

[20] Zeleke LB Welsh A Abeje G Khejahei M. Proportions and determinants of successful surgical repair of obstetric fistula in low- and middle-income countries: a systematic review and meta-analysis. PLoS One. (2024) 19(5):e0303020. 10.1371/journal.pone.0303020 38722847 PMC11081269

[21] Gezimu W Sime T Diriba A Gemechu D. Repair failure and associated factors among women who underwent obstetric fistula surgery in southwest Ethiopia: a retrospective study. Women’s Health. (2023) 19:17455057231192325. 10.1177/17455057231192325 PMC10440064 37596930

[22] Ejigu N Seyoum K Kene C Gomora D Mengistu S Geta G Prevalence and associated risk factors for failed obstetric fistula repair in east African countries: a systematic review and meta-analysis. SAGE Open Med. (2023) 11:20503121231187742. 10.1177/20503121231187742 37492647 PMC10363902

[23] Wanjala A Kipruto H Mabeya H Wang’ombe A Mwang HR. Factors associated with obstetric fistula repair failure among women admitted at gynocare women’s and fistula hospital in Kenya, 2012–2016: a case control study. Nepal J Obstetr Gynaecol. (2019) 13(3):17–23. 10.3126/njog.v13i3.23425

[24] Tadesse S Ejigu N Edosa D Ashegu T Dulla D. Obstetric fistula repair failure and its associated factors among women underwent repair in Yirgalem Hamlin fistula center, Sidama regional state, southern Ethiopia, 2021: a retrospective cross sectional study. BMC Women’s Health. (2022) 22(1):288. 10.1186/s12905-022-01866-z 35811314 PMC9272558

[25] Gessessew A Mesfin M. Obstructed labour in Adigrat zonal hospital, Tigray region, Ethiopia. Ethiop J Health Dev. (2003) 17(3):175–80.

[26] Barone MA Frajzyngier V Ruminjo J Asiimwe F Barry TH Bello A Determinants of postoperative outcomes of female genital fistula repair surgery. Obstet Gynecol. (2012) 120(3):524–31. 10.1097/AOG.0b013e31826579e8 22914460 PMC3437437

[27] Goh JT Browning A Berhan B Chang A. Predicting the risk of failure of closure of obstetric fistula and residual urinary incontinence using a classification system. Int Urogynecol J. (2008) 19:1659–62. 10.1007/s00192-008-0693-9 18690403

[28] Aynie AA Yihunie AG Munae AM. Magnitude of Repair Failure and Associated Factors among Women Undergone Obstetric Fistula Repair in Bahir Dar Hamlin Fistula Center, Amhara Region, Northwest Ethiopia. Brazil: Bibilioteka Virtual Em Saude (2019).

[29] Sori DA Azale AW Gemeda DH. Characteristics and repair outcome of patients with vesicovaginal fistula managed in Jimma University teaching hospital, Ethiopia. BMC Urol. (2016) 16:1–6. 10.1186/s12894-016-0120-3 27406310 PMC4942998

[30] Dereje B Abebe E. Assessment of obstetric fistula and factors associated among women admitted to Jimma Medical Center, south west Ethiopia. Int J Healthc Syst Eng. (2019) 1(003).

[31] Tadesse S Mislu E Tola G Ayenew A Tesfaye M Endeshaw F. Characteristics of patients underwent vesico-vaginal fistula repair and their repair outcomes at Yirgalem Hamlin fistula center, southern Ethiopia. J Women Health Care Gynecol. (2024) 3(5):1–9. 10.59657/2993-0871.brs.24.045

[32] Paluku JL Aksanti BK Clemmer WC Furaha CM Kamabu EM Kasereka JM Determinants and predictive model of failure of surgical repair of obstetric vesico-vaginal fistula in the Democratic Republic of the Congo. Reprod Health. (2024) 21(1):42. 10.1186/s12978-024-01779-0 38561789 PMC10986004

[33] Sjøveian S Vangen S Mukwege D Onsrud M. Surgical outcome of obstetric fistula: a retrospective analysis of 595 patients. Acta Obstet Gynecol Scand. (2011) 90(7):753–60. 10.1111/j.1600-0412.2011.01162.x 21542810

[34] Mafu MM Banze DFK Aussak BTT Kolié D Camara BS Nembunzu D Factors associated with surgical repair success of female genital fistula in the democratic Republic of Congo: experiences of the fistula care plus project, 2017–2019. Trop Med Int Health. (2022) 27(9):831–9. 10.1111/tmi.13794 35749231 PMC9541372

[35] Delamou A Delvaux T Beavogui AH Toure A Kolié D Sidibé S Factors associated with the failure of obstetric fistula repair in Guinea: implications for practice. Reprod Health. (2016) 13:1–9. 10.1186/s12978-016-0248-3 27821123 PMC5100224

[36] McFadden E Taleski SJ Bocking A Spitzer RF Mabeya H. Retrospective review of predisposing factors and surgical outcomes in obstetric fistula patients at a single teaching hospital in western Kenya. J Obstet Gynaecol Can. (2011) 33(1):30–5. 10.1016/S1701-2163(16)34769-7 21272433

[37] Kadra AO. Risk Factors That Contribute to fistula Formation among Women Attending at Borama National Fistula Hospital Somaliland from 2011 to 2014. Kenya: University of Nairobi (2017).

[38] Barageine JK Tumwesigye NM Byamugisha JK Almroth L Faxelid E. Risk factors for obstetric fistula in western Uganda: a case control study. PLoS One. (2014) 9(11):e112299. 10.1371/journal.pone.0112299 25401756 PMC4234404

